# Targeting KIFC1 Promotes Senescence in Soft Tissue Sarcoma via FXR1‐Dependent Regulation of MAD2L1 mRNA Stability

**DOI:** 10.1002/advs.202405611

**Published:** 2024-10-10

**Authors:** Xiu‐Xia Lu, Yi Que, Jing Yang, Li‐Yuan Le, Qi‐Yan Cai, Bu‐Shu Xu, Dong‐Chun Hong, Yao Liang, Xing Zhang

**Affiliations:** ^1^ Melanoma and Sarcoma Medical Oncology Unit Sun Yat‐sen University Cancer Center Guangzhou Guangdong 510060 P. R. China; ^2^ State Key Laboratory of Oncology in South China Guangdong Key Laboratory of Nasopharyngeal Carcinoma Diagnosis and Therapy Guangdong Provincial Clinical Research Center for Cancer Sun Yat‐sen University Cancer Center Guangzhou 510060 P. R. China; ^3^ Department of Pediatric Oncology Sun Yat‐sen University Cancer Center Guangzhou Guangdong 510060 P. R. China; ^4^ Department of Gastric Surgery Sun Yat‐sen University Cancer Center Guangzhou Guangdong 510060 P. R. China

**Keywords:** FXR1, MAD2L1, cellular senescence, soft tissue sarcoma

## Abstract

Patients diagnosed with soft tissue sarcoma (STS) often present at intermediate to advanced stages, with inherently limited therapeutic options available. There is an urgent need to identify novel therapeutic targets. In this study, by screening STS data from the Cancer Genome Atlas (TCGA) and Genotype Tissue Expression (GTEx) databases, KIFC1 is identified as a potential biomarker and a promising therapeutic target for STS. Notably, a significant increase in KIFC1 levels, which exhibited a strong correlation with a poor prognosis in STS patients is observed. The findings revealed that knockout of KIFC1 suppressed STS growth both in vitro and in vivo. Furthermore, KIFC1 is found to regulate cellular senescence in STS, which has not been reported before. that targeting KIFC1 induced cellular senescence via interacting with FXR1, an RNA‐binding protein is discovered, thereby further stabilizing MAD2L1 mRNA in an m6A‐dependent manner. Additionally, the suppression of KIFC1 markedly diminished the growth of patient‐derived xenografts (PDX) and triggered senescence. This study provides the first evidence that KIFC1 inhibition induces cellular senescence through MAD2L1, underscoring KIFC1 as a novel prognostic biomarker and a potential therapeutic target for STS.

## Graphical abstract

This study elucidates the oncogenic role of KIFC1 in soft tissue sarcoma (STS) and provides the first evidence that targeting KIFC1 induces cellular senescence via interacting with FXR1, thereby stabilizing MAD2L1 mRNA in an m6A‐dependent manner. The finding that inhibiting KIFC1 diminishes the growth of STS‐PDX, underscores KIFC1 as a novel prognostic biomarker and a promising therapeutic target for STS.

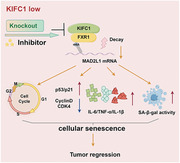

## 1 Introduction

Soft tissue sarcomas (STS) are rare mesenchymal neoplasms that predominantly arise in the trunk, extremities, and retroperitoneum. They account for 1% of adult and 15% of pediatric malignancies, characterized by over 100 different histological subtypes.^[^ ^1^, ^2^ ^]^ Despite the implementation of surgical resection and adjunctive chemotherapy or radiotherapy, approximately 40% of patients experience recurrence or metastasis within 5 years.^[^ ^3^ ^]^ Following the failure of first‐line systemic therapy, alternative treatment strategies include inhibitors that target angiogenesis, immune checkpoints, and specific molecular biomarkers.^[^ ^4^, ^5^, ^6^, ^7^, ^8^ ^]^ Given the limited treatment options available, there exists an urgent need to identify novel potential therapeutic targets.

Kinesin family member C1 (KIFC1, also known as HSET) belongs to the kinesin superfamily (KIFs) and plays a pivotal role in the transport of organelles, proteins, and mRNA complexes using microtubule motors.^[^ ^9^ ^]^ KIFC1 is known for transporting bare double‐stranded DNA,^[^ ^10^ ^]^ a critical process that modulates DNA synthesis.^[^ ^11^ ^]^ Additionally, KIFC1 interacts with other proteins to play a regulatory role in centrosome clustering and chromosomal stability.^[^ ^12^, ^13^, ^14^, ^15^ ^]^ Centrosome amplification is a common feature in numerous tumors,^[^ ^16^, ^17^, ^18^, ^19^ ^]^ and KIFC1, functioning as a regulator of centrosome clustering, was identified as being highly expressed in tumors, significantly contributing to tumor aggressiveness, including treatment resistance, recurrence, and metastasis.^[^ ^20^, ^21^, ^22^, ^23^, ^24^, ^25^, ^26^, ^27^ ^]^ Despite the compelling evidence underscoring KIFC1's notable predictive and potential targeted therapeutic implications in tumors, the expressions and roles of KIFC1 in the pathogenesis of STS remain largely unexplored.

Furthermore, KIFC1 is implicated in centrosome aberrations, with accumulating evidence indicating that centrosome defects foster the acceleration of senescence progression.^[^ ^28^ ^]^ Senescent cells present an enlarged and flattened morphology, exhibit increased activity of senescence‐associated β‐galactosidase (SA‐β‐Gal), activate p16/p53‐dependent cell cycle pathways, and secrete a variety of inflammatory factors.^[^ ^29^ ^]^ Cellular senescence plays a multifaceted and paradoxical role in tumors. While senescent stromal cells and the pro‐inflammatory factors they release can enhance tumor stemness, vascularization, therapeutic resistance, and induce immunosuppression,^[^ ^30^, ^31^, ^32^, ^33^, ^34^, ^35^, ^36^ ^]^ irreversible cell cycle arrest in senescent tumor cells may diminish tumor aggressiveness, increase sensitivity to therapy, activate anti‐tumor immunity, and ultimately facilitate tumor elimination.^[^ ^37^, ^38^, ^39^ ^]^ Therefore, the impact of cellular senescence is contingent upon tumor types and varies in the tumor microenvironment. Recent studies suggest that inducing cellular senescence may offer a new therapeutic strategy for sarcomas,^[^ ^40^, ^41^, ^42^ ^]^ yet the precise mechanisms underlying cellular senescence in STS remain to be fully elucidated.

This study demonstrated the oncogenic role of KIFC1 in STS. Patients with higher KIFC1 expression had poorer prognosis, whereas depletion of KIFC1 impaired STS growth both in vitro and in vivo. Notably, this is the first study to demonstrate that the loss of KIFC1 induced cellular senescence by regulating MAD2L1 expression. Further molecular analyses revealed that KIFC1 interacted with FXR1, which subsequently stabilized MAD2L1 mRNA in an m6A‐dependent manner. Collectively, our findings underscore the significance of KIFC1 in STS and propose that KIFC1 may serve as a novel therapeutic target in STS by promoting cellular senescence.

## 2 Results

### 2.1 KIFC1 Expression is Upregulated in STS and Associated with Poor Outcomes

**Figure 1:**
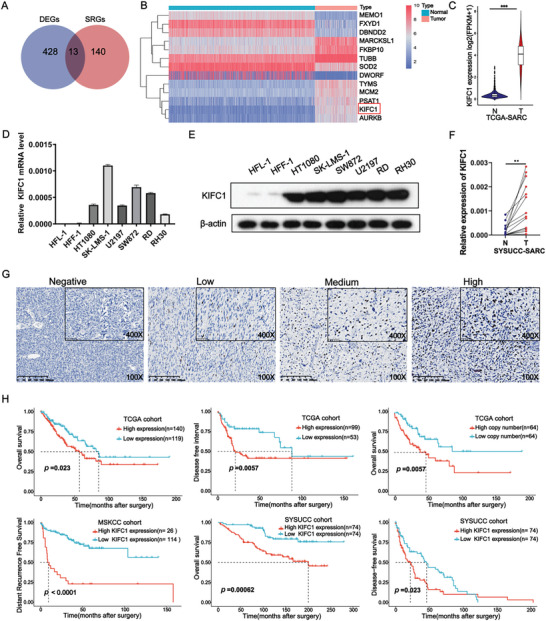
Association of increased KIFC1 expression with poor outcomes in STS. A) Venn diagram illustrating the overlap between differentially expressed genes (DEGs) and survival‐associated genes in STS compared to normal soft tissues. B) Heatmap displaying the expression levels of 13 genes with significant prognostic value. C) Violin plot showing differences in KIFC1 expression levels between STS and normal soft tissues. D, E) Elevated levels of KIFC1 mRNA and protein were observed in STS cell lines, including HT1080, SK‐LMS‐1, U2197, SW872, RD, and RH30. F) Comparison of KIFC1 mRNA levels between STS tissues and corresponding normal tissues, showing increased expression in STS. G) Immunohistochemistry (IHC) images showcasing typical KIFC1 expression in STS tissues. H) Kaplan‐Meier analysis indicating shorter overall survival (OS) and disease‐free survival (DFS) rates in STS patients with higher KIFC1 levels in the TCGA, MSKCC, and SYSUCC cohorts.

Data from the TCGA and GTEx databases were analyzed, identifying the top 441 mRNAs exhibiting the most pronounced differential expression (fold change > 3). These mRNAs were subsequently intersected with the top 153 critical survival‐related genes in STS (*p* < 0.005) (**Figure** **1**A). Thirteen genes met the inclusion criteria of this study. In particular, we focused on KIFC1, based on the exclusion of genes with ambiguous functions and those that had already been extensively researched (Figure 1B). Compared to adjacent normal soft tissues, the expression levels of KIFC1 were elevated in STS, a trend that has also been noted in various other malignancies (Figure 1C; Figure S1A, Supporting Information). Among the various subtypes of sarcoma, leiomyosarcoma (LMS) exhibited elevated transcriptional levels of KIFC1 compared to normal muscle tissue and dedifferentiated liposarcoma (DDLPS) also had higher KIFC1 transcriptional levels than adipocytes (Figure S1B). The expression of KIFC1 in STS cell lines was markedly increased in contrast to HFL‐1 and HFF‐1, which represent human lung fibroblasts and human foreskin fibroblasts, respectively (Figure 1D,E). Additionally, the mRNA levels of KIFC1 were validated to be significantly higher in STS than in corresponding normal soft tissues (Figure 1F). IHC staining was conducted to assess the clinical and prognostic implications of KIFC1 expression in 148 STS patients, with representative graphs illustrating varying histoscore levels displayed in Figure 1G. Correlation analysis revealed a significantly positive association between increased KIFC1 expression and higher tumor grade (*p *< 0.05; **Table** **1**). Furthermore, KIFC1 expression was identified as an independent risk factor for reduced overall survival (OS) in STS patients, as evidenced in both univariate (HR = 3.27, 95% CI = 1.56–6.85, *p* = 0.002) and multivariate analysis (HR = 3.25, 95% CI = 1.34–6.43, *p* = 0.003) (**Table** **2**). Cox regression analysis of KIFC1 levels across 33 types of cancers from the TCGA database demonstrated a correlation between KIFC1 and OS in patients across distinct cancer types (Figure S1C). In the TCGA‐SARC cohort, patients exhibiting higher KIFC1 expression were associated with poorer OS and shorter disease‐free intervals (DFI) (Figure 1H). Additionally, copy number variations of KIFC1 correlated with its expression levels (Figure S1D), with elevated copy numbers indicating a negative prognostic impact (Figure 1H). A similar trend was noted in both the Memorial Sloan Kettering Cancer Center (MSKCC) cohort and our Sun Yat‐sen University Cancer Center (SYSUCC) cohort (*p* < 0.001) (Figure 1H). Collectively, these findings indicate that KIFC1 expression is upregulated in STS and is associated with adverse clinical outcomes.

**Table 1:** Correlation expression of KIFC1 and clinicopathological parameters in 148 STS cases in the SYSUCC cohort.

Characteristic	Total (n=148)	KIFC1 expression		X^2^	*P*‐value
		Low n=74(50%)	High n=74(50%)		
Age (years)					
>50	98	50	48	0.121	0.728
≥50	50	24	26		
Gender					
Male	86	42	44	0.111	0.739
Female	62	32	30		
Tumor grade				3.966	0.046
G1+G2	105	58	47		
G3	43	16	27		
Tumor site				0.036	0.849
Extremity Trunk	111	56	55		
Head/neck & Intra‐	37	18	19		
Histology				4.065	0.254
Fibrosarcoma	49	29	20		
Liposarcoma	46	19	27		
Synovial sarcoma	15	9	6		
others	38	17	21		
Tumor size				0.243	0.622
≤5cm	75	39	36		
>5cm	73	35	38		
Tumor stage				0.275	0.600
I+II	99	51	48		
III+IV	49	23	26		

**Table 2:** Univariate and multivariate analysis of different prognostic parameters in 148 STS patients.

Variable	Univariate analysis		Multivariate analysis	
	HR (95%CI)	p‐value	HR (95%CI)	p‐value
Age (years)				
>50				
≥50	1.61(0.78–3.32)		0.196	
Gender				
Male	1(reference)			
Female	1.18(0.58–2.4)		0.648	
Tumor grade				
G1+G2	1(reference)			
G3	3.05(1.44–6.46)	0.003	1.94(1.05–6.85)	0.167
Tumor site				
Extremity Trunk	1(reference)			
Head/neck & Intra‐abdominal	1.76(0.81–3.82)	0.154		
Histology				
Fibrosarcoma	1(reference)		1(reference)	
Liposarcoma	1(0.39–2.54)	0.992	0.78(0.07–0.48)	0.619
Synovial sarcoma	2.11(0.62–7.14)	0.23	1.96(0.79–10.92)	0.317
others	2.69(1.08–6.72)	0.034	1.67(0.87–8.26)	0.37
Tumor size				
≤5cm	1(reference)			
>5cm	1.73(0.85–3.5)	0.127		
Tumor stage				
I+II	1(reference)			
III+IV	1.47(0.71–3.05)	0.297		
KIFC1 expression				
Low	1(reference)		1(reference)	
High	3.27(1.56–6.85)	0.002	3.25(1.34–6.43)	0.003

### 2.2 KIFC1 Depletion Inhibits the Proliferation and Migration of STS Cells

**Figure 2:**
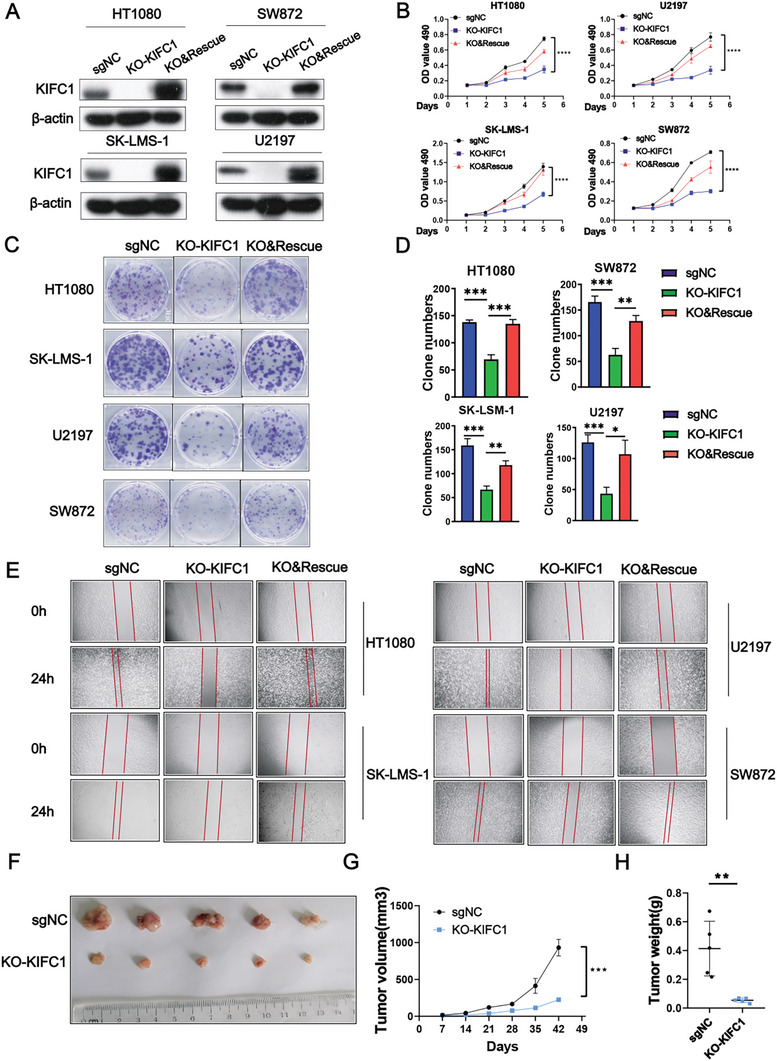
Silencing KIFC1 reduces STS cell proliferation, migration, and tumorigenicity in vitro and in vivo. A) Western blot analysis confirmed complete knockout and subsequent restoration of KIFC1 in specific cell lines. B–D) The impact of KIFC1 on STS cell proliferation and clonogenic potential was evaluated using MTT assays and colony formation tests, respectively. E) KIFC1 knockout (KO) significantly inhibited the migration of STS cells, as shown by wound‐healing assays. F. Representative images of dissected xenografts from HT1080 cells with KIFC1 knockout. G, H) Tumor volume and weight for each experimental group are presented. Error bars represent mean ± SD. ‘ns’ denotes not significant; *p < 0.05; ** *p* < 0.01; *** *p* < 0.001; **** *p* < 0.0001.

To investigate the potential impact of KIFC1 on STS, we developed stable KIFC1 knockout (KIFC1‐KO) and rescue cell lines using SK‐LMS‐1, HT1080, SW872, and U2197(**Figure** **2**A). Given the generally elevated endogenous expression of KIFC1 in STS cells, we did not generate any cell lines with ectopic KIFC1 expression for further functional studies. Knockout of KIFC1 significantly inhibited the growth of STS cells, while restoration of KIFC1 expression effectively reversed these inhibitory effects (*p* < 0.05) (Figure 2B). The colonies formed by STS cells following KIFC1 knockout were both smaller and fewer in number compared to those in the control and rescue groups(*p* < 0.05) (Figure 2C,D). Moreover, the migratory capability of the cells was notably reduced 24 h after KIFC1 knockout, as compared to the control groups (Figure 2E). In vivo experiments showed that mice injected subcutaneously with HT1080/KIFC1‐KO cells developed notably smaller tumors than those in the control group (Figure 2F–H). These findings provide compelling evidence that silencing of KIFC1 inhibits the progression of STS both in vitro and in vivo.

### 2.3 KIFC1 Depletion Induces Cellular Senescence

**Figure 3:**
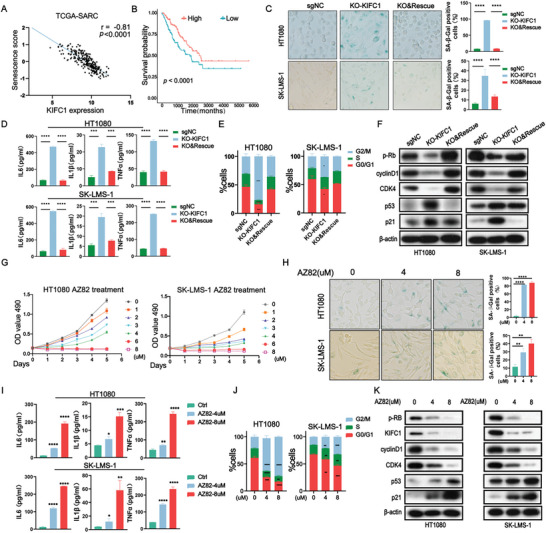
KIFC1 dysfunction triggers cellular senescence. A) Analysis of the correlation between KIFC1 expression and senescence scores in the TCGA‐SARC dataset. B) TCGA‐SARC patients with higher senescence scores exhibited significantly improved overall survival, as shown by Kaplan‐Meier analysis. C) Representative images and quantification of SA‐β‐gal staining, represented as the ratio of SA‐β‐gal‐positive cells to the total number of cells. D) Detection of secreted inflammation‐related factors using ELISA. E) Cell cycle distribution among the G1, S, and G2/M phases. F) Western blot analysis showing levels of p‐Rb, CDK4, cyclin D1, and p53/p21 proteins in SK‐LMS‐1 and HT1080 cells following KIFC1 knockout and subsequent restoration. G) Proliferation rates of STS cells measured by MTT assays after AZ82 treatment. H–K) Analysis of STS cells treated with AZ82, including SA‐β‐gal staining, ELISA, cell cycle analysis, and Western blotting. Error bars represent mean ± SD. ‘ns’ indicates not significant; * *p* < 0.05; ** *p* < 0.01; *** *p* < 0.001; **** *p* < 0.0001.

To further elucidate the biological roles of KIFC1 and to explore other potential mechanisms, we conducted the transcriptome sequencing of KIFC1‐sgNC/KO cells. Gene Ontology (GO) analysis highlighted that KIFC1 influenced processes such as cell cycle arrest, cell death, and cellular senescence (Figure S2A). Additionally, several signaling pathways associated with cellular senescence, including NF‐κB, p53, and TNF pathways, were identified as significantly correlated with KIFC1‐related genes through analysis of the Kyoto Encyclopedia of Genes and Genomes (KEGG) database (Figure S2B). Previous research has indicated that the altered expression of KIFC1 may contribute to cellular senescence.^[^ ^43^ ^]^ To further investigate this, we utilized the TCSER database (http://tcser.bmicc.org/) to analyze the relationship between KIFC1 expression and cellular senescence score. Our analysis revealed a negative correlation between KIFC1 expression and the senescence score (r = −0.81, *p* < 0.0001), indicating that patients with higher senescence scores exhibited better prognosis (*p* < 0.0001) (**Figure** **3**A,B), consistent with previous data.

The role of KIFC1 in inducing cellular senescence was further investigated. The results showed that inhibition of KIFC1 led to senescence in STS cells, as evidenced by an increase in the number of positively stained cells following SA‐β‐Gal staining (Figure 3C). In KIFC1‐depleted cells, there was a notable elevation in the levels of senescence‐associated secretory phenotype (SASP) proteins, including IL‐6, IL‐1β, and TNF‐α (Figure 3D). Moreover, the silencing of KIFC1 resulted in cell proliferation arrest by causing a halt in G2/M of the cell cycle (Figure 3E). Furthermore, the expression of several senescence‐related marker proteins, such as p53 and p21, were significantly increased in KIFC1‐KO STS cells, while the expression of cell cycle‐related proteins, including cyclin D1, CDK4, and p‐Rb were downregulated (Figure 3F).

We then used the KIFC1 inhibitor AZ82 to further investigate the role of KIFC1 in cellular senescence. The results showed that AZ82 effectively inhibited the growth of STS cells in a dose‐ and time‐dependent manner (Figure 3G). Treatment with AZ82 resulted in a senescent phenotype in STS cells, characterized by an increase in SA‐β‐gal‐positive cells, enhanced secretion of inflammatory factors, and a higher proportion of cells arrested in the G2/M phase (Figure 3H–J). Additionally, following AZ82 treatment, KIFC1 protein levels were significantly decreased, and the expression of senescence‐related proteins was notably altered (Figure 3K).

These results reveals that both the knockout and pharmacological inhibition of KIFC1 induce senescence in STS cells.

### 2.4 KIFC1 Enhances MAD2L1 Expression by Stabilizing its mRNA

**Figure 4:**
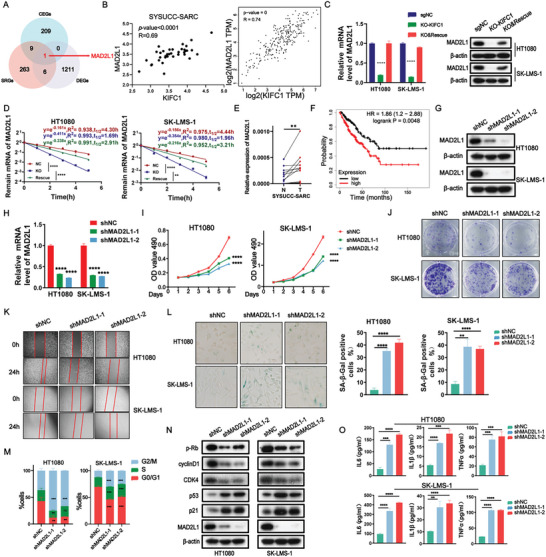
KIFC1 increases mRNA stability of MAD2L1 whose loss of function inhibits the growth and migration of STS cells and induces cellular senescence. A) Venn diagram illustrating the screening process for MAD2L1. B) Linear regression analysis showing the correlation between KIFC1 and MAD2L1 expressions in the TCGA and SYSUCC cohorts. C) Quantitative RT‐PCR and Western blot analysis confirmed changes in MAD2L1 mRNA and protein levels corresponding to KIFC1 levels. D) Assessment of MAD2L1 mRNA stability in KIFC1‐KO STS cells treated with 2 µg/mL actinomycin D over specified time intervals. E) Comparative analysis of MAD2L1 mRNA levels in tumor tissues and paired normal adjacent tissues from the SYSUCC cohort (*n* = 16). F) Kaplan‐Meier analysis evaluating the relationship between MAD2L1 levels and STS prognosis in the TCGA cohort. G, H) Quantitative RT‐PCR and Western blotting were used to measure MAD2L1 mRNA and protein levels in MAD2L1‐knockdown (KD) HT1080 and SK‐LMS‐1 cells. I–K) Effects of shMAD2L1 on STS cell proliferation, colony formation, and migration assessed by MTT, colony formation, and wound‐healing assays, respectively. L. β‐galactosidase staining of MAD2L1‐KD SK‐LMS‐1 and HT1080 cells. M) Cell cycle analysis determining the distribution of cells across G1, S, and G2/M phases. N) Western blot analysis investigating changes in senescence‐related protein levels following MAD2L1 depletion. O) ELISA results showing increased secretion of IL‐6, IL‐1β, and TNF‐α after MAD2L1 knockdown.

To identify the mediator(s) linking KIFC1 deficiency to cellular senescence, we conducted an analysis of the overlap among DEGs following KIFC1 knockout, genes co‐expressed with KIFC1 in the TCGA‐SARC dataset (CEGs), and senescence‐related genes (SRGs) identified from the CellAge database (https://genomics.senescence.info/cells/). Among the identified genes, MAD2L1 emerged as the most significantly associated with KIFC1 in the context of cellular senescence (**Figure** **4**A). We first evaluated the relative expression levels of KIFC1 and MAD2L1 in human STS samples using data from the TCGA and SYSUCC databases, revealing a positive correlation between the expressions of MAD2L1 and KIFC1 (Figure 4B). This correlation was further validated in STS cell lines (Figure 4C; Figure S3A). Subsequently, RNA stability assays indicated that the stability of MAD2L1 mRNA significantly decreased following KIFC1 knockout. Notably, the restoration of KIFC1 expression in KIFC1‐KO cells reversed the MAD2L1 mRNA stability (Figure 4D; Figure S3B).

### 2.5 Inhibition of MAD2L1 Reduces the Growth and Migration of STS Cells and Induces Their Senescence

The functional role of MAD2L1 in tumors and cellular senescence has been documented,^[^ ^44^, ^45^ ^]^ however, its specific role in STS remains largely unexplored and warrants further investigation. Our findings revealed that MAD2L1 expression was significantly upregulated in tumor tissues (Figure S3C). Additionally, univariate Cox regression analyses indicated that high MAD2L1 expression was positively correlated with poorer OS across different types of cancers (Figure S3D). Data from the TCGA‐SARC dataset and SYSUCC cohort showed that MAD2L1 levels were considerably higher in STS tissues compared to normal tissues (Figure 4E; Figure S3E). Patients with STS who exhibited elevated levels of MAD2L1 experienced shorter survival times (Figure 4F). To further investigate the role of MAD2L1, we established stable MAD2L1 knockdown (KD) in HT1080 and SK‐LMS‐1 cell lines and confirmed the efficiency of the knockdown (Figure 4G,H). Knockdown of MAD2L1 resulted in reduced growth, diminished colony formation, and impaired invasion capability in STS cells (Figure 4I–K).

MAD2L1‐KD promoted senescence, as evidenced by an increase in SA‐β‐gal activity in STS cells (Figure 4L) and cells arrested in the S and G2/M phases (Figure 4M). The expression levels of p‐Rb and cell cycle‐associated proteins such as cyclin D1 and CDK4, were downregulated following MAD2L1 inhibition, while levels of p53/p21 were upregulated (Figure 4N). Additionally, MAD2L1‐KD in STS cells also resulted in increased secretion of inflammation‐associated factors (Figure 4O). Collectively, these findings demonstrate that ablation of MAD2L1 plays a crucial role in suppressing STS cell growth, colony formation, and migration, while simultaneously promoting cellular senescence.

### 2.6 MAD2L1 Mediates KIFC1‐Regulated Senescence in STS Cells

**Figure 5:**
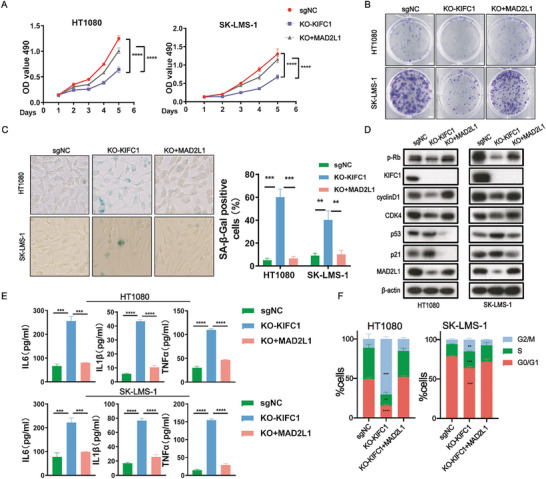
MAD2L1 ectopic expression restores cellular functions altered by KIFC1‐KO. A, B) MTT and colony formation assays were used to assess the effects of MAD2L1 overexpression in KIFC1‐KO STS cells. C) β‐galactosidase staining evaluated the senescence status of KIFC1‐depleted STS cells with or without MAD2L1 overexpression. D) The decreased expression of p‐Rb, CDK4, and cyclin D1, along with the increased p53/p21 expression observed in KIFC1‐KO, were reversed by MAD2L1 ectopic expression, as shown by Western blot analysis. E) ELISA results indicated that MAD2L1 restored the pro‐inflammatory response in KIFC1‐KO cells. F) Cell cycle distribution of STS cells was analyzed by flow cytometry to determine the impact of MAD2L1 overexpression.

To investigate whether MAD2L1 is responsible for the senescence induced by KIFC1 depletion, we overexpressed MAD2L1 in KIFC1‐KO cells. The results showed that ectopic expression of MAD2L1 restored the proliferative and clonogenic capacities of STS cells following KIFC1 knockout (**Figure** **5**A,B). This finding was further supported by SA‐β‐gal staining, which indicated that the proportion of positively stained senescent cells inversely correlated with the overexpression of MAD2L1 in KIFC1‐KO cells (Figure 5C). Ectopic expression of MAD2L1 also reversed the downregulation of cell cycle proteins p‐Rb, CDK4, and cyclin D1, while restoring the upregulation of senescence markers such as p53/p21 (Figure 5D). Additionally, it mitigated the increase in inflammation‐related factors that resulted from KIFC1 silencing (Figure 5E). Furthermore, overexpression of MAD2L1 alleviated the cell cycle arrest induced by KIFC1‐KO and promoted re‐entry into the cell division phase (Figure 5F). In summary, these findings suggest that MAD2L1 plays a crucial role in the KIFC1‐mediated senescence of STS cells.

### 2.7 m6A mRNA Modification is Required for the FXR1‐Dependent, KIFC1‐Mediated Expression of MAD2L1

**Figure 6:**
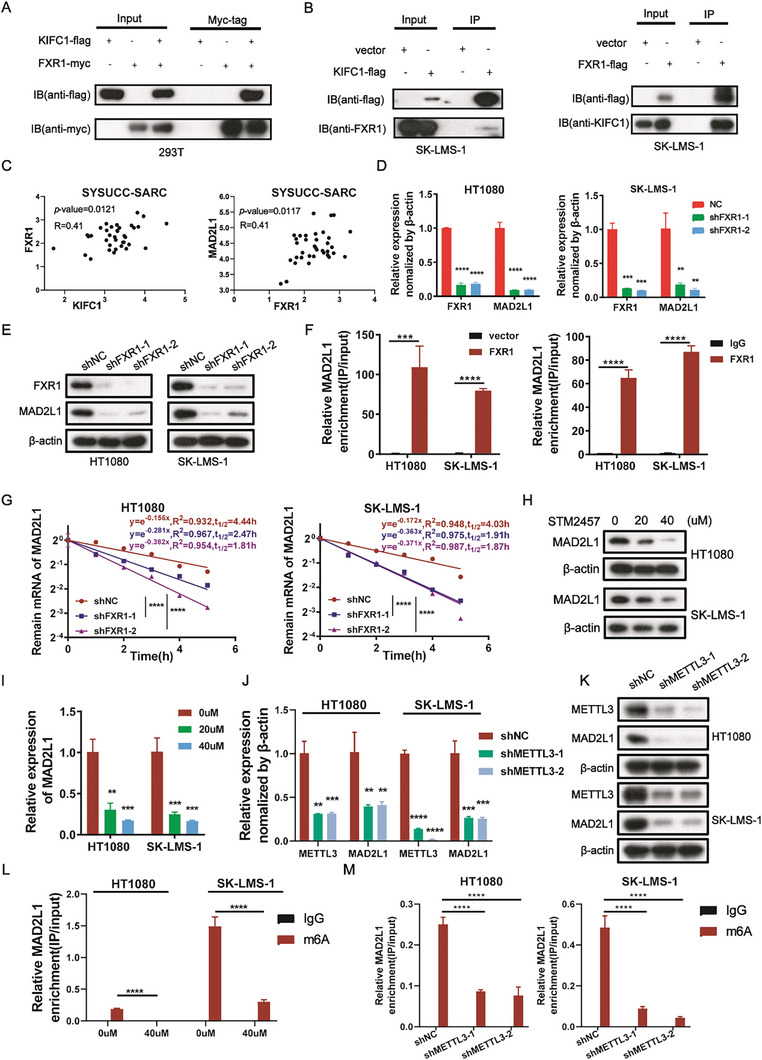
FXR1 mediates the expression of MAD2L1 mRNA in an m6A‐dependent mode. A) A CoIP assay confirmed that KIFC1 interacted with FXR1. B) The lysates of the KIFX1‐Flag or FXR1‐Flag‐transfected SK‐LMS‐1 cells were immunoprecipitated. C) The correlations between KIFC1 and FXR1, as well as FXR1 and MAD2L1, were tested in the 36 specimens. D, E) qRT‐PCR and Western blotting analysis determined the protein and mRNA contents of FXR1 and MAD2L1 in the sh‐ctrl‐ or sh‐FXR1‐transformed HT1080 and SK‐LMS‐1 cells F) RIP‐qPCR was used to examine the relationship between FXR1 and MAD2L1 mRNA G) The decay rate of the MAD2L1 mRNA at the indicated times after exposure of FXR1‐KD STS cells to 5 µg/mL actinomycin D. H, I) Western blotting analysis and qRT‐PCR revealed that STM2457 exposure decreased the MAD2L1 protein and mRNA levels. J, K) METTL3 and MAD2L1 mRNA and protein levels in the sh‐ctrl or sh‐METTL3 SK‐LMS‐1 and HT1080 cells were assessed using qRT‐PCR and Western blots. L, M) The relative m6A‐modified MAD2L1 mRNA levels from SK‐LMS‐1 and HT1080 cells with METTL3‐KD or inhibition were measured by MeRIP‐qPCR.

Given KIFC1's role as a kinesin protein involved in transport, we investigated whether RNA binding proteins (RBPs) interact with KIFC1 to modulate MAD2L1 expression. Screening of RBPs based on previous mass spectrometry results^[^ ^25^ ^]^ indicated that MAD2L1 levels significantly changed following transfection with FXR1 siRNA. The interaction between KIFC1 and FXR1 was confirmed under both exogenous and endogenous conditions through coimmunoprecipitation (coIP) assays (**Figure** **6**A,B), with further validation provided by immunofluorescence assays (Figure S4A,B). Moreover, in human STS samples, we observed a positive correlation between FXR1 and KIFC1 (R = 0.41, *p *= 0.0121; left, Figure 6C) as well as between FXR1 and MAD2L1 expression (R = 0.41, *p* = 0.0117; right, Figure 6C). Following FXR1 knockdown, both mRNA and protein levels of MAD2L1 were significantly downregulated (Figure 6D,E). To further confirm the association between MAD2L1 mRNA and FXR1, we performed RIP assays, which demonstrated that FXR1 bundles to MAD2L1 mRNA (Figure 6F). What is more, FXR1 knockdown (FXR1‐KD) significantly shortened the half‐life of MAD2L1 mRNA (Figure 6G). We then sought to elucidate the mechanism by which KIFC1 regulates MAD2L mRNA. IP‐RIP assays revealed that MAD2L1 mRNA could only be pulled down when both KIFC1 and FXR1 were present (Figure S4C,D). Moreover, when KIFC1 was silenced, FXR1 was unable to bind to and stabilize MAD2L1 mRNA (Figure S4E,F). Collectively, these results indicate that in the presence of both KIFC1 and FXR1, FXR1 binds to and stabilizes MAD2L1 mRNA.

FXR1 has been identified as a novel m6A reader.^[^ ^46^ ^]^ To explore the post‐transcriptional regulation of MAD2L1 expression, we utilized the RM2Target, RMVar, and SRAMP databases, which revealed potential METTL3‐mediated m6A mRNA modification. Treatment of STS cells with STM2457, an m6A inhibitor, resulted in a dose‐dependent decrease in both MAD2L1 mRNA and protein levels (Figure 6H,I). In HT1080 and SK‐LMS‐1 cells, we established a stable METTL3‐KD model using specific shRNAs, which significantly depleted MAD2L1 mRNA and protein levels (Figure 6J,K). Additionally, m6A immunoprecipitation (MeRIP‐qPCR) assays showed a marked decrease in m6A‐modified MAD2L1 mRNAs following disruption or inhibition of METTL3 (Figure 6L,M). In summary, these results indicate that the METTL3‐FXR1 axis enhances the expression and stability of MAD2L1 mRNA in an m6A‐dependent manner.

### 2.8 AZ82 Inhibits STS Progression and Induces Senescence In Vivo

**Figure 7:**
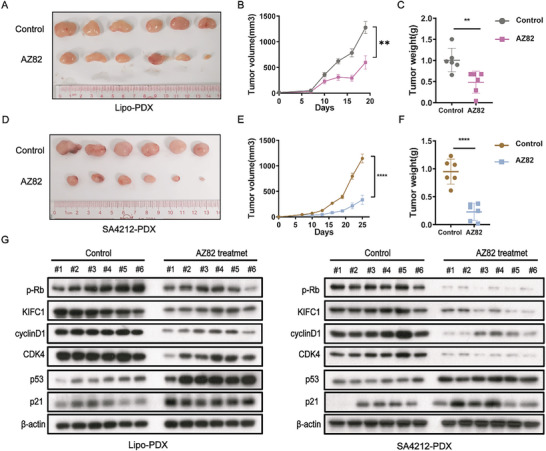
AZ82 prevents STS expansion and induces cellular senescence in vivo. A&D) Photograph illustrating STS PDX treated with vehicle or 50 mg kg^−1^ AZ82 (*n* = 6). B&E) Line plots showing the relative tumor volumes of mice treated with vehicle and AZ82. C&F) Weight of PDX tumors treated with the control or AZ82. G. Western blots illustrated that AZ82 induced senescence in vivo.

Based on the encouraging results from the robust pharmacological inhibition of KIFC1 observed in vitro, we proceeded with subsequent in vivo studies using clinically relevant STS models. Initially, 2 patient‐derived xenografts with distinct genotypes were treated with AZ82 every 3 days. This treatment regimen resulted in impaired engraftment and inhibited the expansion of STS in vivo (**Figure** **7**A–F), without noticeable toxicity or effects on mouse body weight (Figure S4G). Moreover, the levels of the p‐Rb and cell cycle proteins CDK4 and cyclin D1 decreased, whereas the levels of senescence‐associated protein p53/p21 increased, in line with the observations made in vitro (Figure 7G). Collectively, the results indicate that KIFC1 inhibition induces cellular senescence, prompting KIFC1 as a novel prognostic biomarker and a potential therapeutic target for STS.

## 3 Discussion

In addition to the complexities involved in diagnosing, the treatment of advanced‐stage STS presents significant challenges due to the heterogeneity among its various subtypes and the different recommendations for subtype‐specific targeted therapies^[^ ^4^, ^8^ ^]^z In this context, the identification of a universal molecular target that can serve as a prognostic indicator across different histological subtypes, while also providing a foundation for early targeted therapy, is highly sought after. In our screening, KIFC1 emerged as a focus due to its undefined role in STS. Increasing evidence has underscored KIFC1's potential as a prognostic biomarker in various cancers, including hepatocellular carcinoma,^[^ ^20^, ^22^, ^25^ ^]^ esophageal squamous cell carcinoma,^[^ ^24^ ^]^ bladder cancer,^[^ ^21^ ^]^ and prostate cancer.^[^ ^47^ ^]^ Consistent with these findings, our study identified KIFC1 as a novel prognostic biomarker in STS. Through the use of cell assays and a mouse xenograft model, we demonstrated that KIFC1 knockout not only suppressed the growth of STS cells and xenograft tumors but also inhibited STS cell migration in vitro.

In addition to its roles in cell growth and migration, KIFC1 is also crucial in regulating senescence. Over the past decade, the identification of tumor markers has evolved, with cellular senescence now recognized as a hallmark of cancer.^[^ ^48^ ^]^ Increasing evidence suggests that factors such as DNA damage, chromosomal instability, and other stressors can trigger cellular senescence^.[^ ^49^ ^]^ While KIFC1's critical role in maintaining chromosomal stability has been well documented,^[^ ^11^ ^]^ its involvement in cellular senescence has not been extensively studied. Our research represents a pioneering effort in demonstrating that targeting KIFC1 can induce senescence in STS cells, thereby paving the way for novel therapeutic strategies. Utilizing the TCSER database, we confirmed the correlation between KIFC1 expression and cellular senescence. Our investigations also revealed significant alterations in cell cycle changes and inflammatory factors after silencing of KIFC1, which are prominent features of cellular senescence. Specifically, we observed an increase in cell cycle arrest, characterized by a decrease in the levels of p‐Rb/CDK4/ cyclinD1. Conversely, we noted an upregulation of senescence‐associated proteins, including p53 and p21. Certain researchers have suggested that p53/p21 is upregulated during the initial phases of cellular senescence and is reversible, whereas p16 elevates at the terminal stages of cellular senescence. Nonetheless, alternative studies have discovered that p53/p21 or p16 ultimately mediate senescence through p‐Rb.^[^ ^50^, ^51^, ^52^, ^53^ ^]^ Moreover, mouse models established by conditional overexpression of p53^[^ ^38^ ^]^ or p16^[^ ^54^ ^]^ have been employed to investigate the relationship between tumor and cellular senescence. Due to the challenges in detecting p16 in STS cells, we utilized p53/p21 as the representative markers of senescence. Inhibitors targeting KIFC1 have been developed, showing efficacy in blocking tumor growth and metastasis,^[^ ^55^, ^56^, ^57^, ^58^ ^]^ as well as enhancing sensitivity to therapeutic agents.^[^ ^22^, ^25^, ^26^ ^]^ In our study, the KIFC1 inhibitor AZ82 markedly inhibited the growth of STS both in vivo and in vitro, while concurrently promoting senescence.

MAD2L1 plays a crucial role in chromosomal stability^[^ ^59^ ^]^ and enhances the proliferative and migratory capabilities of tumor cells.^[^ ^60^, ^61^, ^62^, ^63^ ^]^ Deficiency in MAD2L1 also leads to cellular senescence.^[^ ^45^, ^60^, ^64^ ^]^ This study provides compelling evidence that loss of MAD2L1 hampered the growth of STS cells and induced senescence. Both KIFC1 and MAD2L1 are involved in DNA synthesis and chromosome segregation during mitosis, and it has been reported that the knockout of either KIFC1 or MAD2L1 alone can independently induce senescence,^[^ ^43^ ^]^ we assume that the mechanisms of inducing senescence are analogical. For the first time, we elucidate the relationship between KIFC1 and MAD2L1. The protein and mRNA levels of MAD2L1 decreased following the knockout of KIFC1, and mRNA stability assays demonstrated that KIFC1 is pivotal in stabilizing MAD2L1 mRNA. Furthermore, we were the first to confirm that ectopic expression of MAD2L1 revered the corresponding function of KIFC1‐KO in inducing senescence. Our results revealed that KIFC1 interacted with the RNA‐binding protein FXR1 to regulate MAD2L1. FXR1 is recognized for its role in promoting malignant behavior across various tumors by stabilizing the mRNA of essential regulators.^[^ ^65^, ^66^, ^67^ ^]^ RIP and mRNA stability assays confirmed the interaction between FXR1 and MAD2L1 mRNA, thereby enhancing its stability. Given FXR1's role as a novel m6A reader,^[^ ^46^ ^]^ we investigated whether MAD2L1 is regulated in an m6A‐dependent manner. Investigations into RNA modifications have revealed that MAD2L1 was subjected to m6A modification, with the m6A RNA methyltransferase METTL3 potentially exerting a regulatory influence. MeRIP‐qPCR results showed that targeting METTL3 through knockdown or inhibition reduced the m6A modification levels of MAD2L1 mRNA. Consequently, these findings unveiled the m6A modification of FXR1 for the first time and demonstrated that KIFC1, via FXR1, regulates MAD2L1 expression by stabilizing its mRNA in an m6A‐dependent manner.

PDX models, formed by implanting tumor tissues from patients into immunocompromised or humanized mice, are widely utilized in drug development, the exploration of drug resistance mechanisms, the prediction of tumor clonal evolution, and various other applications.^[^ ^68^, ^69^, ^70^ ^]^ In this study, we established 2 STS xenograft tumors in BALB/c nude mice, thereby providing a clinically relevant small animal model. The results indicated that the KIFC1 inhibitor AZ82 effectively suppressed STS growth and induced cellular senescence.

In conclusion, KIFC1 was consistently found to be overexpressed in STS and was linked to tumor progression. We were the first to report that targeting KIFC1 diminishes malignancy and induces cellular senescence through the FXR1‐dependent regulation of downstream MAD2L1. However, this study does have limitations, particularly the difficulty of assessing KIFC1's function across more than 100 distinct sarcoma subtypes due to their inherent heterogeneity. Moreover, further research is warranted to elucidate the mechanisms by which AZ82 inhibits tumor progression. Despite these limitations, our study uncovers novel roles of KIFC1 in cellular senescence, underscoring its potential as both a biomarker and therapeutic target for STS.

## 4 Experimental Section

### Cell Culture

For this study, 6 human STS cell lines (HT1080, SK‐LMS‐1, U2197, SW872, RD, and RH30), HEK293T cells, as well as 2 immortalized normal fibroblast cell lines: HFL‐1 and HFF‐1, were used. RH30 was cultured in RPMI 1640 (Gibco) while the other cell lines were cultured in the Dulbecco's modified Eagle's medium (DMEM) (Gibco), supplemented with 10% fetal bovine serum (FBS) (ExCell Bio) and 1% penicillin‐streptomycin. All cell lines were cultured under 5% CO_2_ in a humidified atmosphere at 37 °C.

### Tissue Samples

In total, 16 pairs of STS and adjacent nontumor tissue samples, along with 36 STS tissue samples alone were obtained from the Biological Specimen Bank of the Sun Yat‐sen University Cancer Center in Guangzhou, China, deposited from 2009 to 2018. For immunohistochemical (IHC) experiments, 148 tissue sections embedded in paraffin from patients who had undergone surgery between 2000 and 2018, were collected at the Department of Pathology of the Cancer Center. Prior to the study, all patients provided written informed consent. The Cancer Center's Committee for Ethical Review of Research Involving Human Subjects approved all the samples used.

### Immunostaining

Xylene was used to dewax, and an ethanol gradient was used to dehydrate the tissue samples. Next, the antigen was retrieved using Tris‐EDTA (pH ± 8.0) and incubated in 5% bovine serum albumin (BSA). Finally, the sections were exposed to the anti‐KIFC1 primary antibody (Abcam) overnight at 4 °C and then incubated with the anti‐rabbit goat secondary antibody for 30 min at 37 °C. The proteins were visualized using the DAB solution. IHC staining was analyzed semi‐quantitatively using the H‐score method as described previously.^[^ ^71^ ^]^

### Quantitative Real‐Time PCR

The total RNA was extracted using the TRIzol reagent (Sigma‐Aldrich) and then reverse transcribed into cDNA using the A0010CG Color Reverse Transcription Kit (EZBioscience) according to the manufacturer's protocol. An A0012‐R2 SYBR Green Color qPCR Mix (EZBioscience) was utilized for qRT‐PCR. The mRNA levels were normalized to the levels of β‐actin (ACTB) which was used as an internal control, and the relative expression was calculated using the 2^−ΔΔCt^ method. The primers employed are presented in Table S1 (Supporting Information).

### Plasmid Construction and Transfection

The coding sequences of the human KIFC1 and FXR1, MAD2L1 were PCR amplified and cloned into the pHAGE‐C‐Flag and pHAGE‐C‐Myc lentiviral vectors, with the empty vectors serving as controls. For KIFC1‐knockout (KO), sgRNA targeting it gRNA: TGGGAAGGGGCCTTAATCAG was inserted into the CRISPR/Cas9 All‐in‐One lentiviral vector, followed by cell infection and selection of a single clone. pLKO.1‐puro lentiviral vectors harboring the shMAD2L1#1, shMAD2L1#2, shFXR1#1, shFXR1#2, shMETTL3#1, and shMETTL3#2 shRNA sequences were constructed to knock down these genes (The targeting sequences are shown in Table S2, Supporting Information). For manufacturing the lentiviruses, HEK293T cells were transfected with pMD2.G and psPAX2 (#12259 and #12260; Addgene), the plasmids mentioned above, and PEI (Yeasen) following the instructions of the manufacturer. STS cell lines were then transduced by these viruses. After coinfection for 48 h, 3 µg mL^−1^ puromycin was used to select the cells.

### MTT, Colony Formation, and Wound Healing Assays

To perform the MTT assays, STS cells at a density of 500–1000 cells/well were seeded in 96‐well plates. After cell attachment, the culture medium was added with 20 µL of 5 mg mL^−1^ MTT (Sigma‐Aldrich), incubated at 37 °C for 4 h, and then solubilized. For the color quantification, OD_490_ was measured using an ELX 800 96‐well plate reader (BioTek) on the indicated day (days 1, 2, 3, 4, or 5). The colony formation assays were performed by seeding the cells at 800–1000 cells/well in 6‐well plates, and after 7–10 days of culture, crystal violet was used to stain the colonies that had grown. For the wound healing assays, sterile pipette tips were used to scratch a layer of STS cells when the cell density reached 80%–90% after plating in 6‐well plates. Subsequently, they were cultured in an FBS‐free DMEM medium, and the cell migration distance was recorded at 0 and 24 h.

### Western Blot and Immunoprecipitation Analysis

Whole cells or tissue samples were lysed with 1×sample buffer containing an EDTA‐free protease inhibitor cocktail (TargetMol). Protein lysates were separated on a 9%–12% SDS‐PAGE and transferred to polyvinylidene fluoride (PVDF) membranes. After blocking the membranes with 5% non‐fat milk powder, the primary antibodies were applied and incubated for an entire night at 4 °C. The membranes were washed 3 times with TBST and incubated with the secondary antibody for 1 h at room temperature. Subsequently, the ECL development system was utilized to visualize the protein bands. For immunoprecipitation (IP) assays, the cells were initially rinsed twice with phosphate‐buffered saline (PBS) containing an EDTA‐free protease inhibitor cocktail and kept on ice in preparation. The cells were lysed at 4 °C using IP lysis buffer. Subsequently, 2 µg mg^−1^ of the specific primary antibodies and the supernatant were co‐incubated overnight at 4 °C. The precipitates were washed and boiled to isolate bound proteins. The enriched proteins were separated by SDS‐PAGE and confirmed by Western blotting analysis.

### Immunofluorescence Assay

STS cells cultured in a glass dish were fixed with 4% paraformaldehyde and permeabilized with 0.1% Triton X‐100. After blocking with 5% BSA, the cells were incubated with primary antibodies at 4 °C overnight and then washed with PBS 3 times, followed by incubation with fluorescent secondary antibodies (Invitrogen) for 1 h at room temperature. After washing with PBS 3 times, nuclei were stained with DAPI (Beyotime). Lastly, cells were imaged with a laser scanning confocal microscope (Olympus FV1000).

### RNA Stability Assay

To assess RNA stability, STS cells were seeded into 12‐well plates overnight and treated with actinomycin D (2 µg mL^−1^) for varying periods to inhibit gene transcription. Following isolating the total RNA, qRT‐PCR was performed to examine the gene expression patterns, with normalizing to GAPDH levels.

### RNA Binding Protein Immunoprecipitation (RIP) Assay

Whole cells were washed twice with PBS containing EDTA‐free protease inhibitor cocktail and RNase Inhibitor (MCE), and then lysed with RIP lysis buffer, and 10% of the lysates were collected as input. After adding the primary antibody or IgG with the protein A+G beads (ThermoFisher) or Flag‐beads (Sigma‐Aldrich) to the residual cell lysate, the mixture was incubated on a shaker and left overnight at 4 °C. The non‐specific proteins bound to the beads were released by washing them with the RIP lysis buffer. Similarly, the RNA bound to the beads was extracted by the TRIzol reagent. Then, qRT‐PCR was used to determine the MAD2L1 mRNA enriched by each specific antibody.

### Methylated RNA Immunoprecipitation (MeRIP) Assay

MeRIP‐PCR was used to ascertain the m6A‐modified MAD2L1 mRNA quantitatively. For this, 10 µg of total RNA was extracted and treated with 5 µg of anti‐m6A antibody or IgG at 4 °C overnight. The RNA was then isolated by adding the protein A+G beads, maintaining them at 4 °C for an hour, washing them, and lysing them with a TRIzol reagent. Of these, the enriched m6A‐modified mRNA was detected by qRT‐PCR.

### SA‐β‐Galactosidase Staining for Senescence

The senescence‐associated β‐galactosidase enzyme was assayed by using the C0602 kit (Beyotime), per the instructions. Specifically, the cultivated cells were fixed for 15 min at room temperature in the staining fixative. They were then incubated for 12–24 h at 37 °C without CO_2_ in the working staining solution. The stained cells were examined using an optical microscope and photographed.

### Cell Cycle Analysis

The harvested cells were washed with PBS and fixed overnight in chilled 70% ethanol at −20 °C for the cell cycle analysis. The cells were then rewashed with PBS and treated with the DNA staining solution at room temperature for 30 min. The cell cycle was quantified using an ACEA NovoCyte instrument.

### Tumor Xenograft Model

A subcutaneous xenograft mouse model was established using 6‐week‐old, immunodeficient NCG mice sourced from GuangDong GemPharmatech Company, implanted subcutaneously with HT1080 sgNC/KO cells. Tumor formation was examined by calculating the tumor volume employing the formula V = (width^2^ × length)/2 after 7 days of implantation. All animal experiments were approved by the Ethics Committee of Sun Yat‐sen University Cancer Center (L102012022060A).

### Enzyme‐Linked Immunosorbent Assay (ELISA)

IL‐6, IL‐1β, and TNF‐α levels were quantified by commercial kits. Briefly, the culture medium without FBS was refreshed 8 h before the supernatant was collected. The kit protocols included i) centrifugation to clear the cell debris and ii) adding the reaction buffers to the microplates, followed by incubation before measuring the OD_450_/OD_570_ using ELX 800 96‐well plate reader (BioTek).

### Patient‐Derived Xenograft (PDX) Model

For the human PDX model, 2 cases of STS were enrolled, originating from dedifferentiated liposarcoma (Lipo‐PDX) and synovial sarcoma (SA4212‐PDX), respectively. The P_0_ model was established by subcutaneously transplanting fresh tumor tissues, resected from the Cancer Center's patients between 2020 and 2021, into the BALB/c nude mice obtained from the Animal Center of the Guangdong Medical Laboratory, Guangzhou, China. The P_1_ and P_2_ models were then established once the tumors reached a size of 50–100 mm^3^. The P_2_ mice were grouped into 1) a control group, which received intraperitoneal injections of 100 µL of a solvent (40% PEG‐300, 10% DMSO, 5% Tween‐80, and 45% saline) every 3 days; 2) an AZ82 group, which received intraperitoneal injections of 100 µL AZ82 (MCE) at a dose of 50 mg kg^−1^ every 3 days. The weight and tumor volume of the mice were assessed every 3 days. Once the tumor volume reached approximately 1500 mm^3^, the mice were sacrificed, and the tumors were removed and weighed. Western blotting analysis was performed to detect the senescence‐related proteins.

### RNA Sequencing

TRIzol reagent was used to isolate the total RNA from the STS cells, which were subsequently sequenced at the Berry Genomics Corporation (Beijing, China). Differentially expressed genes, biological pathways, and Gene Ontology (GO) analysis were performed using R software.

### Public Databases

The expressions of KIFC1 and MAD2L1 in the STS and normal tissues were ascertained employing the TCGA and GTEx databases (https://xenabrowser.net/datapages/). The prognostic role of MAD2L1 in STS was identified by the Kaplan‐Meier Plotter (http://kmplot.com/analysed/). The correlation between KIFC1 and MAD2L1, as well as the expression profiles of FXR1, KIFC1, and MAD2L1, were determined using Gene Expression Profiling Interactive Analysis (GEPIA). RM2Target, RMVar, and SRAMP databases were used to obtain information regarding the m6A‐modification of the MAD2L1 mRNA.

### Statistical Analysis

The data were presented as mean ± standard deviation (SD). *p* values < 0.05 were considered statistically significant. The results of any 2 preselected groups were compared using two‐tailed Student's t‐tests accounting for variability. The patients’ survival curve was generated using the Kaplan‐Meier plotter and analyzed by the Log‐rank test. Every parameter identified as significant by the univariate analysis was further subjected to multivariate survival analysis using the Cox regression model. Based on Pearson's correlation coefficients, bivariate correlations between the variables under study were calculated. R software, Adobe Photoshop, Illustratоr, and GraphPad Prism 8.0 were used to create the figures.

### Ethics Approval

The use of human subjects and animals in this study was approved by the Independent Ethics Committee of Sun Yat‐Sen University. Informed consent was obtained from all patients.

## Conflict of Interest

The authors declare no conflict of interest.

## Author Contributions

X.‐X.L., Y.Q., J.Y., L.‐Y.L., and Q.‐Y.C. contributed equally to this work. Conception and design: XZ, XXL. Development of methodology: XXL, YQ, JY. Acquisition of data: XXL, YQ, JY, LYL, QYC. Analysis and interpretation of data: XXL, YQ. Writing, review, and/or revision of the manuscript: XXL, YQ, JY, XZ. Technical or material support: BSX, DCH, YL.

## Supporting information



Supporting Information

## Data Availability

The data that support the findings of this study are available from the corresponding author upon reasonable request.
