# Supplementary material for: Targeting KIFC1 Promotes Senescence in Soft Tissue Sarcoma via FXR1‐Dependent Regulation of MAD2L1 mRNA Stability
Source: Adv Sci (Weinh). 2024 Oct 10;11(44):2405611. doi: 10.1002/advs.202405611 (PMC11600285; doi:10.1002/advs.202405611)

Targeting KIFC1 promotes senescence in soft tissue sarcoma via FXR1-dependent regulation of MAD2L1 mRNA stability

**Supplementary figures and tables**


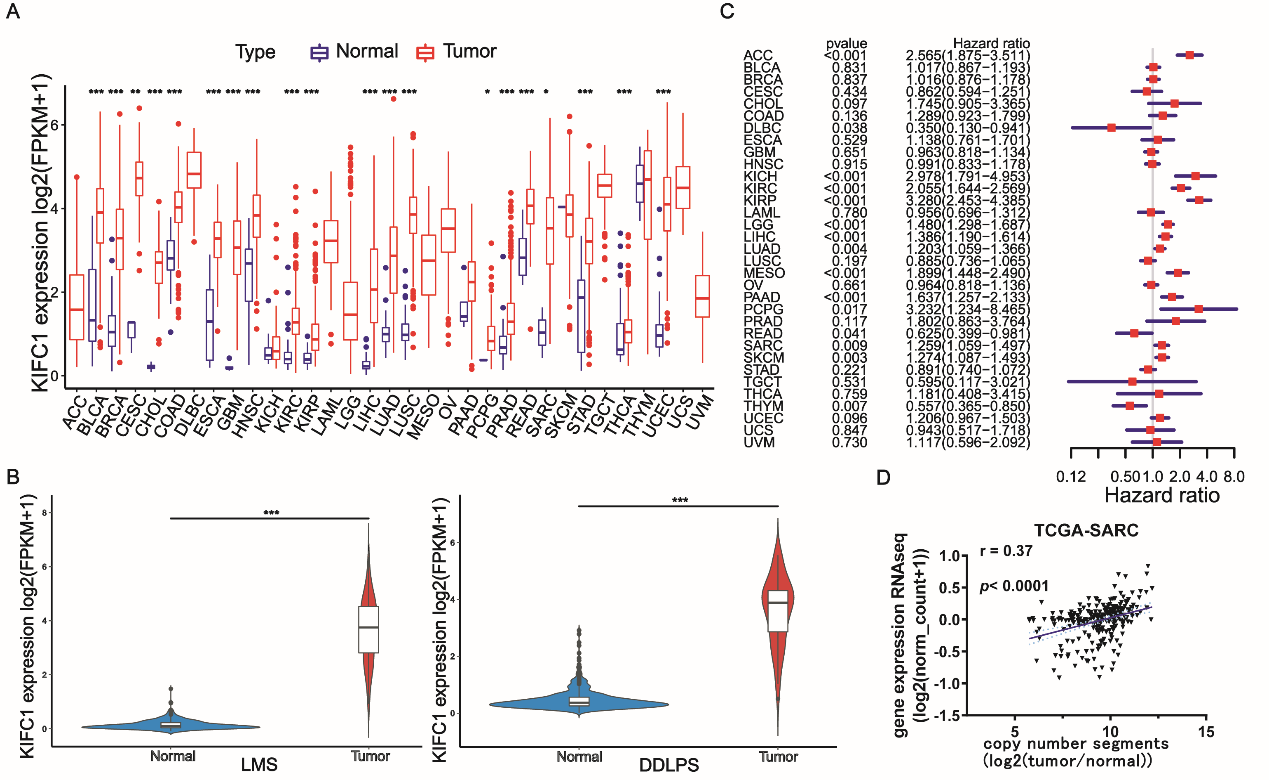


**Supplementary Fig. 1 Increased KIFC1 expression in pan-cancer.**

A. KIFC1 expression in tumor and normal tissues in pan-cancer data of The Cancer Genome Atlas (TCGA).

B. KIFC1 expression in leiomyosarcoma (LMS), dedifferentiated liposarcoma (DDLPS) and corresponding normal tissues.

C. A forest plot of hazard ratios of KIFC1 in 33 types of tumors.

D. The correlation between KIFC1 copy numbers and gene expression in TCGA-SARC.


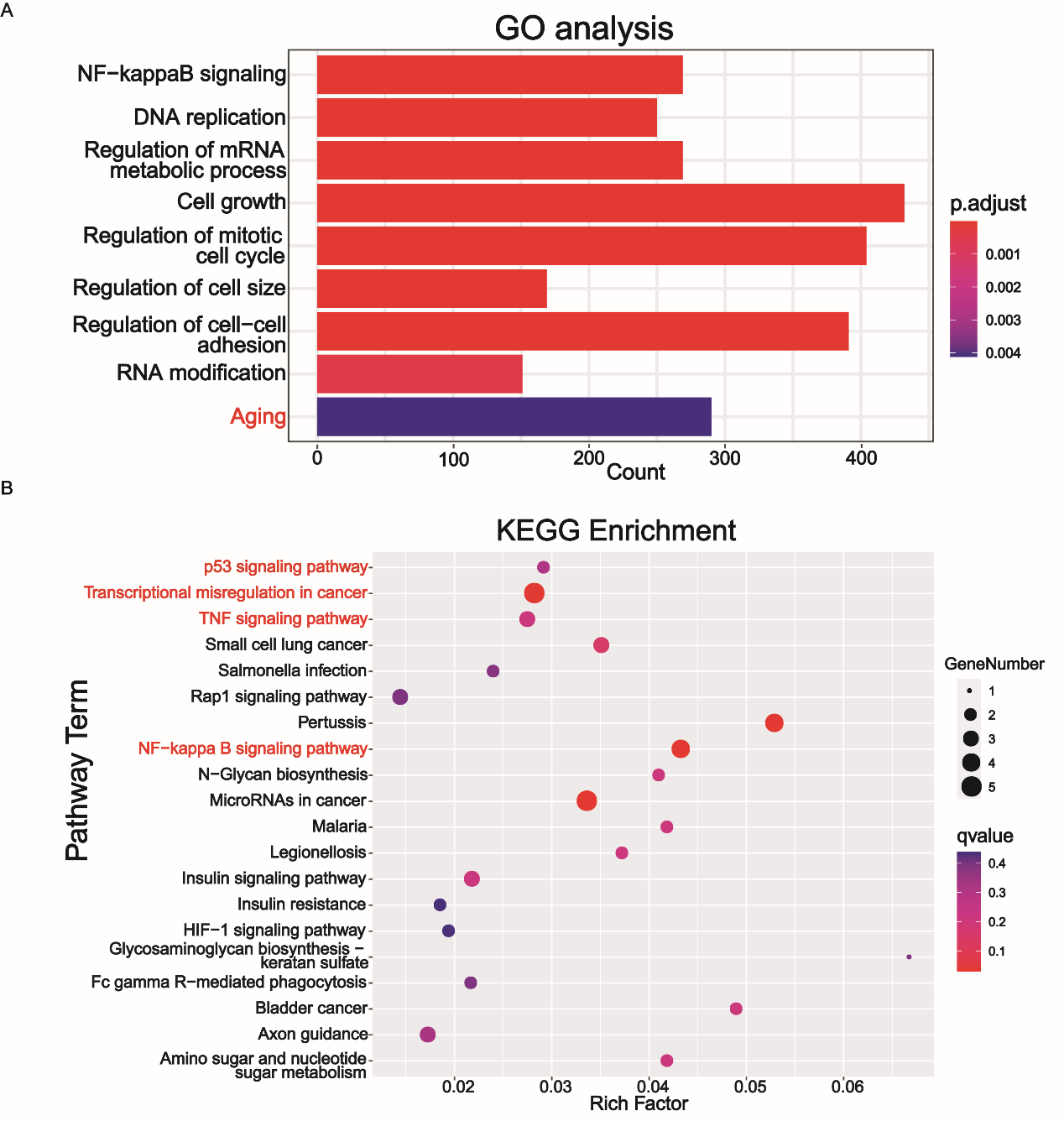


**Supplementary Fig. 2 Knocking out KIFC1 is associated with cellular senescence pathway.**

1. Significant Gene Ontology terms of differentially expressed genes associated with KIFC1, including biological processes.
2. KEGG pathways involved.


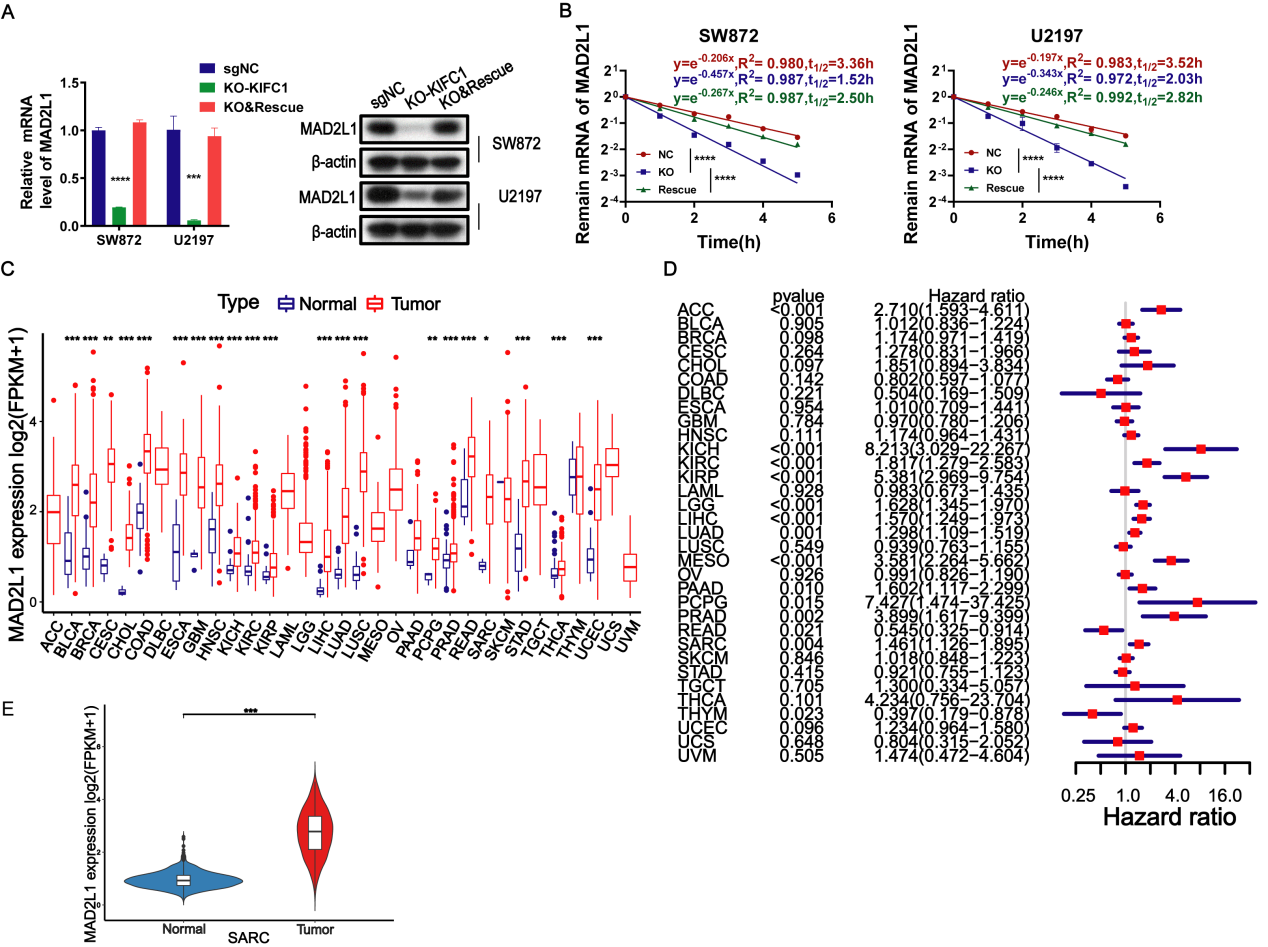


**Supplementary Fig. 3**

1. qRT-PCR and Western blot analysis showed that the mRNA and protein level of MAD2L1 changed with KIFC1 consistently.
2. The mRNA levels of MAD2L1 in KO-KIFC1 STS cells treated with actinomycin D (2 μg/mL) at the indicated time points.
3. MAD2L1 expression in tumor and normal tissues in pan-cancer data of The Cancer Genome Atlas (TCGA).
4. A forest plot of hazard ratios of MAD2L1 in 33 types of tumors.
5. Comparison of the expression of MAD2L1 in soft tissue sarcoma and normal tissues from TCGA and GTEx database.


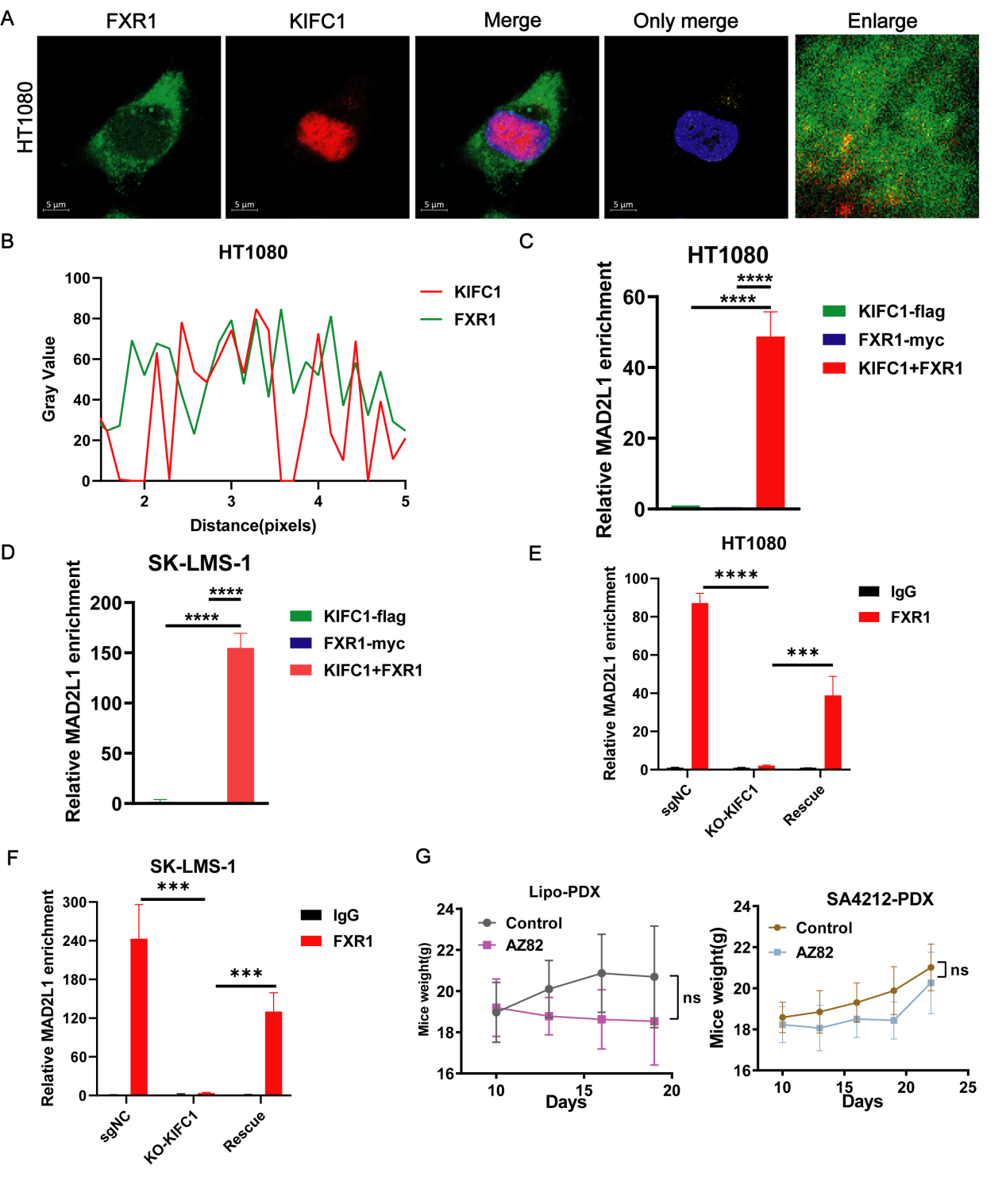


**Supplementary Fig. 4**

1. IF assay demonstrated the colocalization of KIFC1 and FXR1 in HT1080.
2. Plot profiles of KIFC1 and FXR1 intensities in enlarged immunofluorescence image. Red curve shows the gray value of KIFC1, green curve shows the gray value of FXR1.

C-D. IP-RIP assays confirmed the interaction between KIFC1, FXR1 and MAD2L1 mRNA.

E-F. RIP assays were used to examine the role of KIFC1 in FXR1-binding MAD2L1 mRNA.

G. Mice bodyweight changes for the Lipo-PDX /SA4212-PDX treated with vehicle and AZ82.


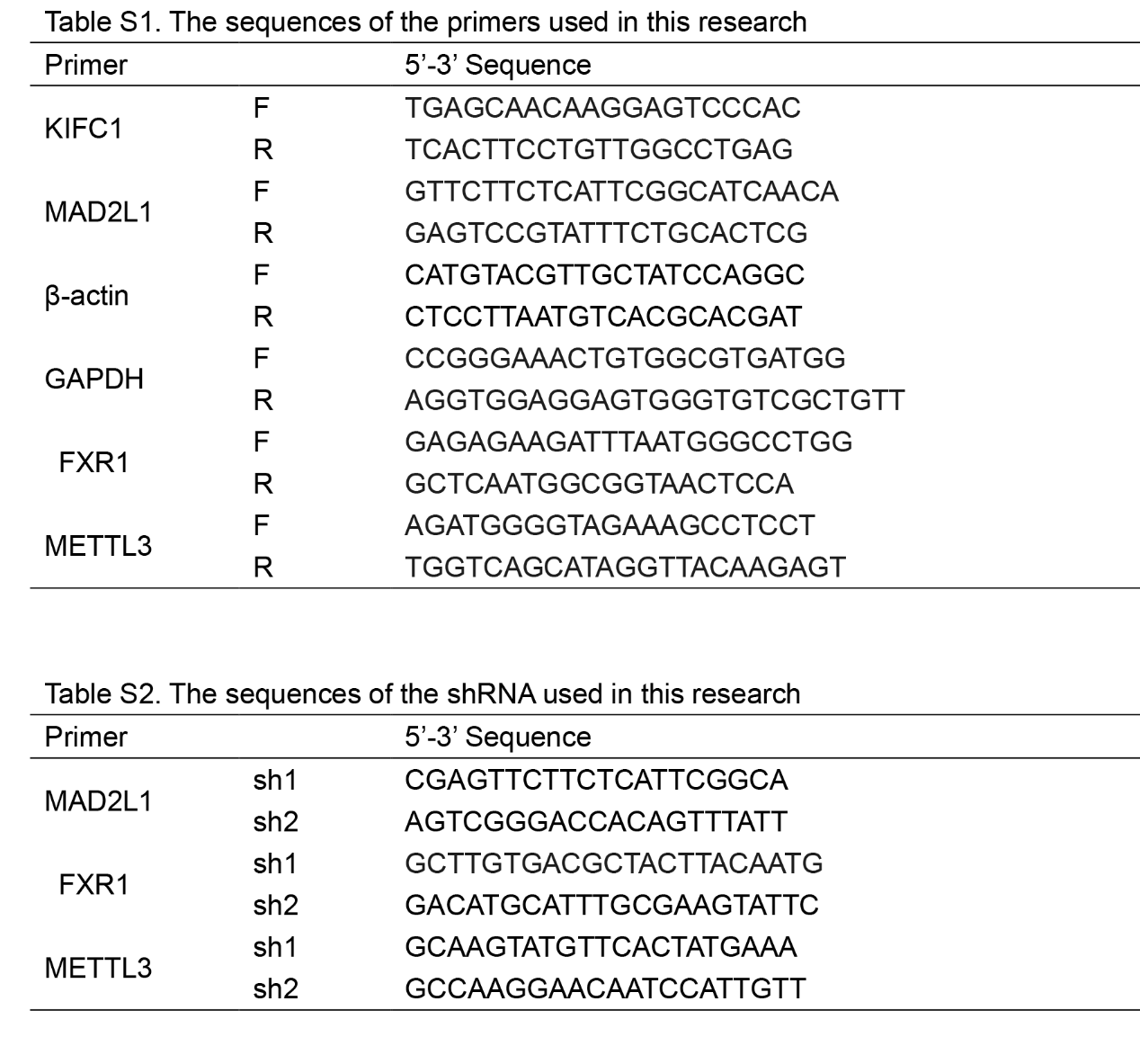

Supplement: Supplementary file 1 — Supporting Information [file ADVS-11-2405611-s001.docx]
